# Prevalence and Predictors of HIV among Chinese Tuberculosis Patients by Provider-Initiated HIV Testing and Counselling (PITC): A Multisite Study in South Central of China

**DOI:** 10.1371/journal.pone.0089723

**Published:** 2014-02-21

**Authors:** Junjie Xu, Weiming Tang, Shiming Cheng, Tanmay Mahapatra, Lin Zhou, Yuji Lai, Yongjun Jiang, Feiying Liu, Xinan Zhen, Jinge He, Jing Zhang, Jinxin Lu, Sanchita Mahapatra, Hong Shang

**Affiliations:** 1 Key Laboratory of AIDS Immunology of National Health and Family Planning Commission Department of Laboratory Medicine, The First Affiliated Hospital, China Medical University, Shenyang, Liaoning, China; 2 Collaborative Innovation Center for Diagnosis and Treatment of Infectious Diseases, Hangzhou, China; 3 Department of Epidemiology, Fielding School of Public Health, University of California, Los Angeles, California, United States of America; 4 Center for Tuberculosis Control and Prevention, National Center for Disease Control and Prevention (CDC), Beijing, China; 5 Center for Tuberculosis Control and Prevention, Guangxi Autonomous Region CDC, Nanning, China; 6 Center for Tuberculosis Control and Prevention, HenanProvincialCDC, Zhengzhou, China; 7 Center for Tuberculosis Control and Prevention, SichuanProvincialCDC, Chengdu, China; University of Rochester, United States of America

## Abstract

**Background:**

Tuberculosis (TB) and HIV are two worldwide public health concerns. Co-infection of these two diseases has been considered to be a major obstacle for the global efforts in reaching the goals for the prevention of HIV and TB.

**Method:**

A comprehensive cross-sectional study was conducted to recruit TB patients in three provinces (Guangxi, Henan and Sichuan) of China between April 1 and September 30, 2010.

**Results:**

A total of 1,032 consenting TB patients attended this survey during the study period. Among the participants, 3.30% were HIV positive; about one quarter had opportunistic infections. Nearly half of the participants were 50 years or older, the majority were male and about one third were from minority ethnic groups. After adjusting for site, gender and areas of residence (using the partial/selective Model 1), former commercial plasma donors (adjusted OR [aOR] = 33.71) and injecting drug users(aOR = 15.86) were found to have significantly higher risk of being HIV-positivity. In addition, having extramarital sexual relationship (aOR = 307.16), being engaged in commercial sex (aOR = 252.37), suffering from opportunistic infections in the past six months (aOR = 2.79), losing 10% or more of the body weight in the past six months (aOR = 5.90) and having abnormal chest X-ray findings (aOR = 20.40) were all significantly associated with HIV seropositivity (each p<0.05).

**Conclusions:**

HIV prevalence among TB patients was high in the study areas of China. To control the dual epidemic, intervention strategies targeting socio-demographic and behavioral factors associated with higher risk of TB-HIV co-infection are urgently called for.

## Introduction

Being the second largest death-causing infections globally (after HIV), tuberculosis (TB) still remains a major public health concern [1]. According to the Global Tuberculosis Report, 2012, published by World Health Organization (WHO), the number of new TB infection was estimated to be 8.7 million worldwide in the year 2011, and about 1.4 million people unfortunately died from this curable disease in the same year. Geographically, Asia and Africa had the highest burden, while India and China together accounted for almost 40% of the total TB cases globally [1].

The dynamics of the epidemic of TB are considered to be driven partially and modified considerably by the HIV epidemic [2], [3]. Among the 8.7 million incident cases that occurred in 2011, about 13% were found to be co-HIV-positivity [1].Additionally, the cumulative reported HIV/AIDS cases (343 thousands) only accounted for less than half of the estimated number of national people who live with HIV(PLWHs, 780 thousands), high proportion of PLWHs failed to know their HIV infection status [4]. Previous studies demonstrated that HIV was one of the main reasons that lead to the failure of TB control programs in countries having high HIV burden [5], [6], [7]. Clinical and epidemiological research has established that active TB in HIV-1 infected subjects is commonly associated with increased HIV-1 related immunodeficiency and mortality [8]. Co-infection of these two diseases has been observed to culminate into a major obstacle for the global efforts in reaching the goals for the prevention of HIV and TB [2].

Alike some other countries, China is also experiencing the menace of HIV-TB co-infection with reported HIV prevalence ranging from 0.1% to 8.4% among TB patients [9]. Prior studies conducted here has demonstrated that the individual prevalence proportions of HIV and TB within mainland China are considerably high and the co-infections of these two are also common, posing a serious threat to the respective disease control programs and wellbeing of people in this most populous country of the world [9].

Studies conducted so far to develop insights into the issue were not robust enough to conduct a precise and externally valid estimation of the overall burden of these co-infections owing to small sample size and limited geographical distributions. Although some of these studies explored the interaction between these two diseases, potential predictors of HIV infection among TB patients remained mostly understudied, thus further limiting the effectiveness of the control programs in minimizing co-infection [8], [10].

The complicated nature of the dual epidemic and the interplay called for a detailed quantification of the disease burden and identification of important risk factors so that targeted interventions with specific focus can be designed and strategized to curb the epidemics. To conduct a precise and externally valid quantification of the problem of TB-HIV co-infection and to identify important socio-behavioral correlates of HIV infection among TB patients, a comprehensive survey was conducted in three provinces of China to provide evidences for future strategies to design efficient programs for the prevention of these diseases.

## Methods

### HIV Epidemic of the Study Sites

At the end of 2011 the number of accumulative reported HIV/AIDS cases of Guangxi, Henan and Sichuan Province ranked the second, the third and the fourth among all the national provinces/autonomous regions/municipalities, respectively [4].

### Participant Recruitment

All the eligible, consenting TB patients from four counties in three provinces of China (Nanning in Guangxi, Wolong and Zhenping in Henan and Dazhu in Sichuan) were recruited for the survey at the tuberculosis control institutes. All the selected counties had the largest number of reported TB patients among respective provinces, while HIV epidemic was less reported among TB patients of these selected counties. The inclusion criteria included: 1) aged 18 years or more, 2) agreed to participate and provided informed content, and 3) met the diagnostic criteria for TB. Convenient sampling was used to recruit all the TB patients who attended TB clinics/hospitals in the selected counties for the first time between April 1 and September 30 in the year 2010. Eligible subjects already recruited for the study were not included again in their further visits during the study period.

### Demographic and Behavioral Measures

After collecting the written informed consent, a face-to-face questionnaire based interview was conducted by trained TB physicians from the study sites for each of the eligible participants to collect information on socio-demographics, profile as a TB patient, HIV related behaviors and opportunistic infections. Socio-demographic information included: age (under 30 years, 30 to 49 years, 50 years and above), gender (male or female), race (Han or others), marital status (never married, currently married, divorced/widowed), education (elementary school and below or junior high school and above), areas of residence (urban or rural), official residency (resident of one of the cities under study or resident of a city other than the cities under study) and annual income (3000 Yuan and less or more than 3000 Yuan). Information on profile as a TB patient included: body mass index (BMI, it was calculated by TB patients reported weight and height; 18.5 and below or more than 18.5), reason for attending TB clinics/hospital (came to the TB clinics/hospital by themselves, referred from other hospitals or other reasons including: recommended by private practitioners/close contacts of TB patients/came for routine health examination/other medical problems), disease (TB) category (new onset or recurrent), sputum smear test result (not tested, tested negative for TB or tested positive for TB) and chest X-ray report (abnormal/normal). Enquired HIV related behaviors were: being former commercial plasma donors (yes/no), being engaged in injecting drug use (yes/no), history of extramarital sex (yes/no) and HIV status of the regular sexual partner (no regular partner, do not know, negative or positive). Participants who experienced symptoms like: oral thrush, continuing diarrhea, signs of atypical respiratory infections or visual impairment during past six months, were classified as having opportunistic infections. Weight loss was defined as 10% or more decrease in body weight during past six months.

### Serologic Measures

Trained physicians prescribed HIV antibody testing by using the Provider-Initiated HIV Testing & Counselling (PITC) strategy for all eligible TB patients attending their TB control institutes, unless participants declined to test HIV or self-reported had HIV positive testing result before.

For HIV testing, 3 ml of intravenous blood was collected from each of the eligible and consenting subjects for HIV testing. HIV antibodies were screened using ELISA test (Acon Biotech Co., Ltd). If the screening result was positive, the diagnosis was confirmed by Western Blot test (HIVBLOT 2.2, Genelabs Diagnostics, Singapore).TB was confirmed by looking at a sputum smear under a microscope for those TB patients who were consented and could provide smear specimens. To detect any radiological discernible lung finding, chest X-Ray film exam was conducted for each of the participants. HIV antibody screening test, sputum smear and chest X-Ray film exam were conducted at the selected tuberculosis control institutes of the four selected counties. And HIV antibody confirming test was implemented at the AIDS labs of three above local provincial center for disease control and prevention (CDC).

### Statistical Analysis

Data entry was done ensuring accuracy by double entry and multiple logic checks using EpiData 3.1 [11]. SAS statistical analysis software version 9.1 [12] was used for the statistical analyses. Initially descriptive analyses were conducted to describe the distribution of socio-demographic factors, participants’ profile as TB patient, behaviors known to be associated with risk of HIV-positivity, opportunistic infections and percentage positivity of HIV among the TB patients of the provinces under study. Simple logistic regression procedure was used next to conduct univariate analysis [odds ratio (OR) and 95% confidence intervals (95% CI)] to determine individual association of socio-demographic, clinical and behavioral factors with HIV sero-positivity status among TB patients of the above-mentioned provinces. Then we conducted multiple logistic regression method using two separate models (Model 1 and 2) to estimate the above mentioned association adjusted for potential confounders (as identified in the contextual scientific literatures). In the partial/selective model of multivariable analysis (Model 1) sites, genders and areas of residence were included as confounders while in the full/saturated model (Model 2) age and annual income were adjusted additionally.

### Ethical Aspects

After explaining the details of the study, potential benefits and risks associated with it, written informed consent was obtained from each of the subjects prior to the interview and blood collection. The study protocol was developed by the first affiliated hospital of China Medical University, and approved by Ethics Committee of No.1 Hospital of China Medical University(number 2008 [73]) in late 2008.

## Results

This study was conducted between the months of April and September in the year 2010. During this period, altogether 1,032 consenting TB patients who met the inclusion criteria were recruited. Among the participants, 57.66% (595) were recruited from Nanning cityin Guangxi province, 9.40% (97) from Wolong and 23.26% (240) from Zhenping County in Henan province while 9.68% were recruited from Dazhu county in Sichuan province. Nearly half (47.67%) of the participating patients belonged to the age group of 50 years or more, the majority (68.99%) were male and about one third (31.20%) of the study subjects were from minority ethnic groups. Approximately three quarters (74.81%) were married, 62.21% had junior high school or higher level of education, proportion of urban residents (52.33%) was higher than rural, most of the patients (88.86%) were resident of the study areas and more than half of them (50.48%) reported to have annual income of 3000 Yuan or less(see Table 1).

**Table 1:** Socio-behavioral and clinical characteristics along with HIV sero-positivity among participating tuberculosis (TB) patients in three provinces of China, 2010 (N = 1032).

Variable		Number	Percentage
Site (TB Clinics/Hospitals)	Nanning in Guangxi province	595	57.66
	Wolong in Henan province	97	9.40
	Zhenping in Henan province	240	23.26
	Dazhu in Sichuan province	100	9.68
Age(years)	≤30	245	23.74
	30 to 49	295	28.59
	≥50	492	47.67
Gender	Male	712	68.99
	Female	320	31.01
Race	Han	710	68.80
	Other	322	31.20
Marital status	Never married	217	21.03
	Currently married	772	74.81
	Divorced/widowed	43	4.17
Education	Elementary and below	390	37.79
	Junior high school and above	642	62.21
Areas of residence	Urban	540	52.33
	Rural	492	47.67
Officially resident of	One of the cities under study	917	88.86
	Cities other than the cities under study	115	11.14
Annual income	≤3000 Yuan	521	50.48
	>3000 Yuan	511	49.52
Body mass index(BMI index)	18.5 and below	310	30.04
	>18.5	722	69.96
Reason for attending TB hospital	Came to the TB hospital by themselves	430	41.67
	Referred from other hospitals	476	46.12
	Other reasons	126	12.21
Disease (TB) category	New onset	998	96.71
	Recurrent	34	3.29
Sputum smear test result	Not tested	811	78.58
	Negative for TB	69	6.69
	Positive for TB	152	14.73
Chest X-Ray test	Abnormal	1019	99.03
	Normal	10	0.97
Former commercial plasma donors	Yes	12	1.16
	No	1020	98.84
Injecting drug users	Yes	10	0.97
	No	1022	99.03
Extramarital sex	Yes	26	2.52
	No	1006	97.48
HIV status of regular sexual partner	No regular partner	196	18.99
	Do not know	811	78.59
	Negative	25	2.42
	Positive	0	0.00
Opportunistic infections	Yes	249	24.13
	No	783	75.87
HIV	Positive	34	3.30
	Negative	998	96.70

BMI of 30.04% recruited TB patients were found to be 18.5 or lower, 46.12% were transferred from other hospitals, the majority of them (96.71%) had new onset infections, 6.69% had sputum smear test negative for TB and almost all the participants (99.03%) had abnormal lung findings detected in the chest X-ray(see Table 1).

Regarding the potentially high risk (for HIV-positivity) behaviors, 1.16% of the participants were former commercial plasma donors, 0.97% of them had history of injecting drug use (IDU), 2.52% reported to have ever been engaged in extramarital sex and 78.59% of the participants did not know the HIV status of their partners. About one quarter (24.13%) of the subjects reported to have any kind of opportunistic infections. 34 participating TB patients (3.30%) were found to be HIV positive in these four counties(see Table 1).

The results of the univariate analysis (presented in Table 2 under the column heading “Crude”) indicated that alcoholism was significantly associated with HIV seropositivity (OR: 2.05, 95% CI: 1.01–4.16) with reference to the non-alcoholics. Among the participating TB patients, former commercial plasma donors (OR: 16.51, 95% CI: 4.71–57.84) and IDUs (OR: 22.04, 95% CI: 5.91–82.21) had significantly higher odds of HIV seropositivity compared to the non-donors and non-IDUs respectively. Unadjusted analyses also revealed that: being involved in extramarital sex (OR: 235.87, 95% CI: 82.23–676.58), history of commercial sex (OR: 248.50, 95% CI: 75.60–816.86), suffering from opportunistic infections in the past six months (OR: 2.27, 95% CI: 1.13–4.57), having lost 10% or more of the body weight in the past six months (OR: 4.66, 95% CI: 2.20–9.85), having positive sputum smear test results for TB (OR: 3.65, 95% CI: 1.24–10.70) and abnormal chest X-ray findings (OR: 22.76, 95% CI: 6.09–85.02 ) were all associated with significantly higher risk of HIV-positivity among the study subject, compared to the respective reference groups(see Table 2).

**Table 2:** Factors correlated with HIV infection among TB patients in three provinces of China, 2010 (N = 1032).

Variable		Crude		Model 1*	Model 2#
		OR	95% CI	aOR(95% CI)	aOR(95% CI)
BMI	*>18.5*	Ref		Ref	Ref
	*≤18.5*	1.12	0.54–2.32	1.08(0.51–2.30)	0.94(0.43–2.02)
Smoking	*No*	Ref		Ref	Ref
	*Yes*	1.44	0.72–2.86	1.04(0.49–2.22)	1.13(0.52–2.45)
Alcoholism	*No*	Ref		Ref	Ref
	*Yes*	2.05	1.01–4.16	1.53(0.72–3.26)	1.80(0.82–3.96)
Former commercialplasma donors	*No*	Ref		Ref	Ref
	*Yes*	16.51	4.71–57.84	33.71(6.76–168.00)	39.14(7.87–194.59)
Injecting drug users	*No*	Ref		Ref	Ref
	*Yes*	22.04	5.91–82.21	15.86(3.97–63.34)	11.49(2.72–48.51)
Extramarital sex	*No*	Ref		Ref	Ref
	*Yes*	235.87	82.23–676.58	307.16(86.01->999.99@)	272.56(75.24–987.29)
Commercial sex	*No*	Ref		Ref	Ref
	*Yes*	248.50	75.60–816.86	252.37(66.69–955.09)	224.85(58.81–859.63)
Opportunistic infections	*No*	Ref		Ref	Ref
	*Yes*	2.27	1.13–4.57	2.79(1.34–5.83)	3.17(1.49–6.75)
Weight loss	*No*	Ref		Ref	Ref
	*Yes*	4.66	2.20–9.85	5.90(2.66–13.07)	6.02(2.73–14.08)
Sputum smear results	*Negative*	Ref		Ref	Ref
	*Positive*	3.65	1.24–10.70	2.51(0.80–7.89)	2.71(0.84–8.80)
	*Not tested*	0.76	0.30–1.96	0.57(0.21–1.57)	0.57(0.20–1.60)
Chest X-ray	*Normal*	Ref		Ref	Ref
	*Abnormal*	22.76	6.09–85.02	20.40(4.64–89.68)	26.58(5.83–121.21)

*Model 1 adjusted for site, genders and areas of residence.

#: Model 2 adjusted for sites, genders, areas of residence, age and annual income.

@: The model did not work well.

Boldfaced figures indicated statistically significant results at α = 0.05.

After adjusting for sites, genders and areas of residence (using the partial/selective Model 1), alike the univariate analyses, former commercial plasma donors (OR: 33.71, 95% CI: 6.76–168.00) and injecting drug users (OR: 15.86, 95% CI: 3.97–63.34) were found to have significantly higher risk of being HIV-positivity with reference to the non-donors and non-IDUs respectively. Similar to the unadjusted analyses, having extramarital sexual relationship (OR: 307.16, 95% CI: 86.01- >999.99, although the model did not work well here due to sparse data), being engaged in commercial sex (OR: 252.37, 95% CI: 66.69–955.09), suffering from opportunistic infections in the past six months (OR: 2.79, 95% CI: 1.34–5.83), losing 10% or more of the body weight in the past six months (OR: 5.90, 95% CI: 2.66–13.07) and having abnormal chest X-ray findings (OR: 20.40, 95% CI: 4.64–89.68) were all significantly associated with HIV seropositivity among the participating TB patients(see Table 2).

Using the full/saturated model (Model 2) for multivariable logistic regression analyses, where we additionally adjusted for the age of the patient and annual income, the results did not change much compared to the Model 1(see Table 2).

## Discussion

In this comprehensive survey involving a diverse group of TB patients from four counties in three provinces of China, the sero-positivity of HIV was found to be 3.30%. This observed sero-positivity level among TB patients was much higher than the National HIV prevalence (0.058%) of China [13] and observed HIV prevalence among TB patients in studies conducted during 2005–07 at Guangxi (0.5%) [14], Shandong (0.1%) [15]and Hebei (0.1%) [16]. Except the findings from one study in Xinjiang during 2002 (4.5%) and another in GungXi during 2006 (4.3%) all other studies included in a Meta analysis on HIV/TB Co-Infection in Mainland China revealed lower percentage positivity of HIV among TB patients compared to our observation [9].

Being conducted simultaneously at multiple provinces of the country, our study was likely to be based on a diverse and potentially representative sample. The observed percentage positivity may be considered as a more realistic picture of the HIV epidemic among TB patients in the selected provinces, necessitating urgent target-oriented disease control programs among TB patients in China. It also highlighted that PITC was a good strategy to find HIV infection cases in medical environment and this strategy should be expanded for China to find more hidden PLWHs.

In this study, participation of the TB patients belonging to minority groups was considerably high (31.20%). The potential reason for this distribution was probably the fact that about two third of the participants were recruited from the two provinces (Guangxi and Sichuan) which had larger percentages of minorities. Especially in Guangxi, minority groups accounted for about one fifth of the total minority population in the mainland China.

About half of the recruited TB patients were from rural areas and more than one third of them were either illiterate or attended elementary schools only. The combination of these two observations might have indicated a potential lack of awareness regarding health and availability of healthcare services along with poor health seeking behavior. All these taken together in turn might well translate into increased risk of acquisition of infectious disease, including TB and HIV [17]. Only 11.4% participants were not the official residents of the city from where they were recruited, which probably meant that the majority of this study population were not migrants from other places and thus the efficient roll out of disease control programs among them might not be extremely difficult [18].

In our study, only 2.52% subjects reported to have ever been engaged in extramarital sex. This observed proportion was less than the findings of a study conducted among former commercial plasma donors in rural areas of China [19] and another involving the employed male rural migrants in Shanghai (both studies report a percentage of about 6%) [20]. One possible explanation for this result could be the fact that about half of the participants were recruited from rural and poor areas of China where the common Chinese culture of mostly monogamous sexual relationships are still practiced largely [21]. The possibility of having reporting or social desirability bias in the self-reported information could also be another possibility.

Based on their reported signs or symptoms, about 24% of the participants of this study were diagnosed to have some opportunistic infections. This finding corroborated with observations from previous studies where infections with mycobacterium tuberculosis were found to be associated with inadequate immune response of the host [22]. This immune-suppression among the TB patients could also have led to the increased susceptibility to many kinds of opportunistic infections [23].

While estimating the magnitude and direction of association between risk of HIV infection and their potential socio-demographic and behavioral correlates, TB patients who were former commercial plasma donors or IDUs seemed to have much higher likelihood of being HIV sero-positive with respect to their non-donor and non-IDU counterparts respectively. This finding corroborated with observations from several previous studies [19], [24], [25] and might easily be explained by the fact that HIV epidemic in China was once significantly driven by HIV spread through these two direct routes of transmission while commercial plasma donors and IDUs were two population subgroups with extremely high risk of HIV-positivity in China [19], [26].

Alike several other studies conducted previously [27], [28], participants reporting the history of being ever engaged in commercial sex were observed to have much higher odds of contracting HIV infection compared to those who reported to have no such exposure. Based on the available information in the contextual background literature, unprotected vaginal intercourse (UVI) with commercial sex workers might be suspected as the main contributory behavior regarding this finding, because UVI had been found to be a common practice among TB patients who had exposure to commercial sex and it is one of the well-established risk factors for the HIV-positivity [29], [30].

Suffering from some opportunistic infection was associated with higher propensity of being HIV positive among the participating TB patients in this study. The disease of tuberculosis itself might have played an important role in this associational pathway, by inducing immune-suppression among the TB patients and thus making them more vulnerable to both HIV and other opportunistic infections [31], [32]. Potential explanations might also include the probability that being HIV-positivity might have resulted in immune-suppression and thus leading to higher likelihood of harboring TB and other opportunistic infections among the participants [33]. However, due to the inherent issue of temporal ambiguity in a cross-sectional study, our survey results could not claim to have identified any distinction between these two above mentioned possibilities and further studies with longitudinal design were required to identify the actual direction of the associational pathway with more certainty. Either way it seemed to be of paramount importance to have targeted intervention programs in place focusing on the control of opportunistic infections among TB patients to minimize the pathos of the dual epidemic of TB and HIV in China. Corroborating with the findings from prior studies [34], significant weight loss in the past six months was also found to be positively associated with risk of HIV infection among TB patients in our study.

Both the crude and adjusted model did show that radiologically discernible abnormal chest findings among TB patients were significantly associated with HIV seropositivity. To the best of our knowledge, no study had so far reported this relationship, and thus the potential reasons are still unclear. Immune-suppression in co-infected patients might have resulted in more advanced stage of active TB leading to radiologically identifiable lung findings.

According to our knowledge, this was the first effort in China to determine the strength and direction of association between the risk of HIV infection and its potential predictors among TB patients in a large, diverse and somewhat representative study population. By recruiting patients from multiple cities in three different provinces of the country, our study was able to conduct the research involving a diverse sample of TB patients having widespread geographical distribution. The measured burden of HIV and the observed associations of the risk of HIV-positivity with its potential predictors can thus have a somewhat larger scope of extrapolation to guide the policy-makers while designing appropriate targeted interventions. Large sample size, use of biological markers, advanced laboratory investigation techniques and following uniform study protocol aided by extensive training of all study personnel to minimize interviewer bias were major strengths of this study.

As an observational study, our study had several limitations. As we discussed before, being based on the self-report of the participants, our study was vulnerable to suffer from reporting and social desirability bias. Although the resulting exposure and confounder misclassifications were more likely to be non-differential as the HIV testing results were conveyed after the completion of the interviews, the possibility of differential misclassification could not be ruled out completely as some of the participating TB patients might already have the symptoms of HIV or the diagnosis made earlier [35]. We still consider the size of this information bias to be small, based on the assumption that while attending the clinics/hospitals for seeking healthcare services, our study subjects, being all TB patients were likely to be mostly truthful. As indicated before, because of the cross-sectional design of our study, in built temporal ambiguity prevented us from drawing any causal inferences based on our results and we recommend that any such interpretation should be made with caution. Consenting TB patients who attended TB clinics/hospitals in the selected counties during the study period were recruited for the study. If non-participation of TB patients were influenced by the study variables and their HIV sero-positivity status then selection bias might be an issue in the results our study. Although we do not have any information regarding the non-participants, considering the strength of coverage of the TB surveillance program of China, we don’t expect the potential magnitude of this bias to be much bigger. Despite of having a relatively large overall sample size, in our study only 34 patients were tested positive for HIV, 12 subjects stated that they were former commercial plasma donors, 10 participants reported to be injecting drug users and 26 people reported to have ever been engaged in extramarital sex. All of these smaller numbers might have resulted in sparse data problems, leading to the observed lack of precision in our results as exemplified by the width of the 95% CIs for the estimated ORs in the study. This lack of power might also have led to the problem of residual confounding, as the sample size issue limited our ability to control for all the measured potential confounders. Alike any other observational study the possibilities of having some unmeasured or unknown confounding of the results were also there.

To minimize the potential for information bias, all of the study personnel who conducted the face-to-face interviews were rigorously trained using uniform guideline across all the sites and the same study protocol was followed at every study sites. To assure consistency and completeness of the collected data, designated persons at each study sites checked every individual questionnaire before the participants left the sites. Upon detection of any logical error or incompleteness, the interviewees had the opportunity to correct them before leaving.

## Conclusions

Even with certain limitations, we can still conclude that the HIV prevalence among TB patients was high in the above-mentioned provinces of China. To control these dual epidemic, intervention strategies targeting socio-demographic and behavioral factors associated with higher risk of TB-HIV co-infection are urgently called for. These targeted intervention programs need to focus high risk population groups especially on those who ever had extramarital sex, were former commercial plasma donors or injecting drug users and those who are suffering from any opportunistic infection.
